# Enhanced active case finding of drug-resistant tuberculosis in Namibia: a protocol for the hotspots, hospitals, and households (H3TB) study

**DOI:** 10.1136/bmjopen-2023-082665

**Published:** 2024-02-10

**Authors:** Olga Shavuka, Etuhole Iipumbu, Lorraine Boois, Gunar Günther, Graeme Hoddinott, Hsien-Ho Lin, Emmanuel Nepolo, Stefan Niemann, Nunurai Ruswa, James Seddon, Mareli M Claassens

**Affiliations:** 1 Department of Human Biological and Translational Medical Sciences, University of Namibia, Windhoek, Khomas, Namibia; 2 Inselspital, University of Bern, Bern, Switzerland; 3 Desmond Tutu TB Centre, Department of Pediatrics and Child Health, Stellenbosch University Faculty of Medicine and Health Sciences, Cape Town, Western Cape, South Africa; 4 National Taiwan University, Taipei, Taiwan; 5 Molecular and Experimental Mycobacteriology Group, Forschungszentrum Borstel, Borstel, Germany; 6 National Tuberculosis and Leprosy Programme (NTLP), Windhoek, Namibia; 7 Department of Infectious Disease, Imperial College London, London, UK

**Keywords:** Tuberculosis, Epidemiology, Public health, Diagnostic microbiology

## Abstract

**Introduction:**

Namibia is a high tuberculosis (TB)-burden country with an estimated incidence of 460/100 000 (around 12 000 cases) per year. Approximately 4.5% of new cases and 7.9% of previously treated TB cases are multidrug resistant (MDR) and 47% of patients with MDR-TB are HIV coinfected. Published data suggest a clustering of MDR-TB transmission in specific areas. Identifying transmission clusters is key to implementing high-yield and cost-effective interventions. This includes knowing the yield of finding TB cases in high-transmission zones (eg, community hotspots, hospitals or households) to deliver community-based interventions. We aim to identify such transmission zones for enhanced case finding and evaluate the effectiveness of this approach.

**Methods and analysis:**

H3TB is an observational cross-sectional study evaluating MDR-TB active case finding strategies. Sputum samples from MDR-TB cases in three regions of Namibia will be evaluated by whole genome sequencing (WGS) in addition to routine sputum investigations (Xpert MTB/RIF, culture and drug susceptibility testing). We will collect information on household contacts, use of community spaces and geographical map intersections between participants, synthesising these data to identify transmission hotspots. We will look at the feasibility, acceptability, yield and cost of case finding strategies in these hotspots, and in households of patients with MDR-TB and visitors of hospitalised patients with MDR-TB. A compartmental transmission dynamic model will be constructed to evaluate the impact and cost-effectiveness of the strategies if scaled.

**Ethics and dissemination:**

Ethics approval was obtained. Participants will give informed consent. H3TB will capitalise on a partnership with the Ministry of Health and Social Services to follow up individuals diagnosed with MDR-TB and integrate WGS data with innovative contact network mapping, to allow enhanced case finding. Study data will contribute towards a systems approach to TB control. Equally important, it will serve as a role model for similar studies in other high-incidence settings.

**STRENGTHS AND LIMITATIONS OF THIS STUDY**

- State-of-the-art sequencing techniques are implemented and used in a low-income and middle-income country setting at the University of Namibia (UNAM).
- Novel drug-resistant tuberculosis (DR-TB) case finding strategies are piloted and evaluated for feasibility, acceptability, yield and cost.
- The study is implemented in three Namibian regions which would make the findings generalisable; the transmission model will inform on the impact of scale-up of the case finding strategies in the whole country.
- Existing partnerships between the research group, the National TB and Leprosy Programme (NTLP), the Research Center Borstel and the Ministry of Health and Social Services, could be leveraged to support implementation.
- The study team depends on an existing partnership between UNAM and the NTLP/National Institutes of Pathology to obtain sputum culture samples for sequencing; if the routine collection and evaluation of sputum samples do not happen systematically, DR-TB cases might be missed.

## Introduction

Namibia is classified by the WHO as a high tuberculosis (TB)-burden country and, in 2020, the TB incidence was estimated to be 460/100 000, with 12 000 new cases/year.^1^ The most recent nationwide anti-TB drug resistance survey in Namibia was completed in 2015, reporting a prevalence of multidrug-resistant (MDR)-TB at 4.5% among new cases and 7.9% among previously treated cases. Approximately 46.6% of patients with MDR-TB were coinfected with HIV.^2^ Based on that prevalence, the number of MDR-TB cases/year is estimated to be around 800. Only 213 cases were detected in 2020.

Namibia faces significant challenges at every step along the TB care cascade, from preventive therapy to diagnostics and patient treatment. Challenges also exist in the identification of prevalent cases of TB infection and disease through passive and active case finding intervention strategies.^3^ During the COVID-19 pandemic, Namibia observed an increased number of missed or undiagnosed TB cases with case management interruptions due to the diagnostic supply chain, redirections of resources and the effects of human movement restrictions, negatively impacting care-seeking.^4^

In the ‘hotspots, hospitals, and households’ (H3TB) study, we aim to identify MDR-TB transmission clusters, through genomic, geospatial and social data interferences, which could indicate transmission hotspots. We aim to determine the diagnostic yield of targeting these areas for active case finding as well as evaluating the cost-effectiveness of such approaches. Our central hypothesis is that geographical heterogeneity in the concentration of genotypes exists within the MDR-TB epidemic in Namibia leading to high-transmission areas (hotspots), where MDR-TB incidence could be up to three times that of the baseline population. Further, if these areas could be identified and targeted through enhanced case finding strategies, these strategies might be more effective than screening and treatment programmes which do not adapt to spatial and biological variation.^5^ In addition to targeting transmission hotspots, our focus will be on MDR-TB screening of household contacts of MDR-TB cases and visitors of hospitalised patients with MDR-TB.

## Methods and analysis

### Patient and public involvement

We have collaborated directly with the National TB and Leprosy Programme (NTLP) (Dr Nunurai Ruswa, coauthor) on the development of this protocol and with the national reference laboratory, the National Institutes of Pathology (NIP) (Ms Queen Mokomele). Since we are a young research group, we have not yet instituted community advisory boards (CABs) to discuss potential studies with community members. However, in a subsequent study, we have funding and the expertise to establish a CAB. The University of Namibia (UNAM) research ethics committees will incorporate community members from 2024 onwards.

### Design

**Figure 1:**
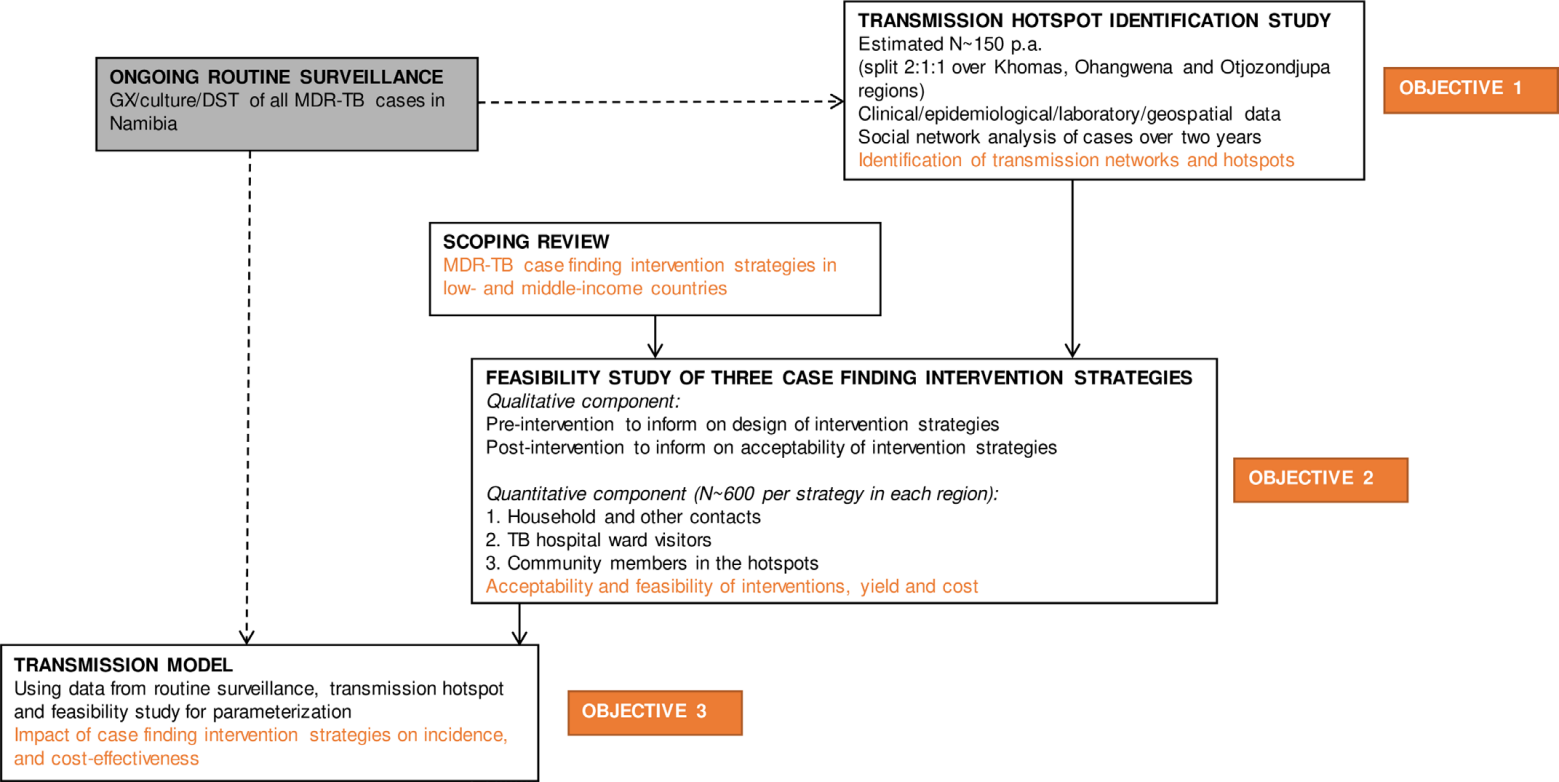
Flow diagram of H3TB Study design. Black text indicates methods; orange text indicates outcomes. DST, drug sensitivity testing; H3TB, hotspots, hospitals, and households; MDR-TB, multidrug-resistant TB; p.a., per annum; TB, tuberculosis.

This is an observational study including the design and evaluation of case finding intervention strategies to determine feasibility. The study has three objectives (table 1). In figure 1, the study flow is illustrated. Among an ongoing parent surveillance study which includes all MDR-TB cases in Namibia from all regions, H3TB is nested within three regions (Khomas, Otjozondjupa and Ohangwena). These regions are known to have a high TB incidence. In areas in the Otjozondjupa region, the case notification rate has been >1000/100 000 (submitted for publication, Claassens). The ongoing parent surveillance study informs the H3TB research team on potential participants to include in objective 1. In addition, a scoping review looks at existing MDR-TB case finding strategies in low-income and middle-income countries (LMICs) and informs objective 2. Data from the surveillance study and the first two objectives inform the development of the dynamic transmission model, which is objective 3.

**Table 1:** Objectives of H3TB Study

Objective	Activity
1	To identify transmission hotspots using routine data from the National TB and Leprosy Programme, whole genome sequencing, geospatial and social data from the research study
2	To conduct a feasibility study of three active case finding interventions in three geographical regions
3	To develop a dynamic transmission model to determine the impact of scaled-up interventions on MDR-TB incidence and evaluate cost-effectiveness

H3TB, hotspots, hospitals, and households; MDR-TB, multidrug-resistant TB; TB, tuberculosis.

### Setting

The study will be conducted in Namibia in three regions, viz., Khomas, Ohangwena and Otjozondjupa. Recruitment has commenced in August 2022 and will continue until December 2024.

### Objective 1

#### Participant identification

Individuals with a positive Xpert MTB/RIF result with rifampicin resistance demonstrated, and a positive *Mycobacterium tuberculosis* culture, will be included from the three regions. To identify transmission hotspots, whole genome sequencing (WGS) results will be combined with clinical, epidemiological, geospatial and social network data. A traditional sample size calculation is not indicated if most of the MDR-TB cases in a region are included (>80%).^5 6^ In this study, we might miss some MDR-TB cases who are not reported or diagnosed. However, a current surveillance study which started in January 2020 and is ongoing could inform on additional participants from these areas. We will look at other strategies, like including participants from other regions or finding additional cases through collaborating with the NTLP’s active case finding interventions, to ensure robustness of our data.

#### Data collection

Primary data will be collected using standardised questionnaires. We will use a novel approach of first engaging the participants in an in-depth qualitative interview with participatory data collection activities to facilitate them explaining their family, household, social and community circumstances. These in-depth qualitative data will facilitate the data collectors’ ability to confirm details, probe further and mitigate a bias toward the under-reporting of contacts. As part of the questionnaires, we will capture a single Global Positioning Satellite (GPS) coordinate for each participant’s home and other places in the community which will be identified as ‘shared spaces’ on electronic devices. We will record the coordinate of a participant’s home when the field team is at his/her home or at a community space. A separate enrolment form will be used to capture names and other identifying information. Data from the transmission hotspot identification study will be linked to electronic TB register data through personal identifying information such as name, surname and date of birth. A social network questionnaire will be used to identify shared socialisation settings, with GPS coordinates of these settings recorded.

#### Laboratory analyses

DNA will be isolated from sputum cultures at the NIP biosafety level 3 laboratory and the UNAM laboratory for samples with proven GeneXpert MTB/RIF rifampicin resistance and shipped to Research Center Borstel (RCB) for WGS. Isolated genomic DNA of individual strains will be sequenced using Illumina sequencing platforms and Nextera library preparation kits. All isolates will be sequenced with a minimum coverage of 50-fold. Raw read data (fastq files) will be mapped to the *M. tuberculosis* H37Rv genome (GenBank accession number NC_000962.3) using BWA-MEM, and mappings will be refined with the GATK software package. For variant (InDels and single nucleotide polymorphisms (SNPs)) detection in mapped reads, custom perl scripts will be employed with thresholds of a minimum coverage of four reads in both forward and reverse orientation, four reads calling the allele with at least a Phred score of 20 and 75% allele frequency. Multiple consecutive SNP calls (in a 12 bp window) that could indicate rare recombination sites or reflect artificial variant calls around InDels will be excluded. For alignment of a concatenated sequence for downstream analyses, variants in drug resistance-associated genes and repetitive regions will be excluded. Phylogenetic lineages (*M. tuberculosis* genotypes and known Beijing subgroups) will be inferred from specific SNPs.^7 8^ As proxy for TB cases associated with direct transmission events, a maximum pairwise genetic distance between at least two *M. tuberculosis* isolates of five SNPs will be used.^9^

#### Data management and analysis

Data will be captured into databases developed by a data specialist. WGS data will be analysed using a phylogenetic strain classification and a cluster/similarity analysis. Social network data will be analysed using thematic analysis for the description of typical and atypical social network contacts for people with MDR-TB in Namibia. Similarly, thematic analysis will be used for shared space data to identify the types of places where people with MDR-TB interact with other people to whom TB may be transmitted. Social network analysis, in conjunction with WGS cluster data, will indicate transmission hotspots within the regions. In our context, a hotspot is an area with a high TB incidence and where TB transmission happens, based on microbial genomic methods such as WGS and a collection of epidemiological approaches (social network and spatial analysis) and geographical map intersections between participants.

### Objective 2

#### Participant identification

For the feasibility study, participants will be enrolled from three zones, namely (a) transmission hotspots, (b) household and other close contacts of MDR-TB cases identified by the TB programme, and (c) visitors to patients with MDR-TB admitted to TB wards in hospitals. A sample size calculation is not indicated since one of the goals of feasibility studies is to inform on sample size calculations for the planning of large-scale appropriately powered intervention studies.^10 11^ A sample size of 600 participants for each intervention strategy will inform on the feasibility and acceptability of the interventions, the yield and the cost per additional case.

#### Data collection

There will be both qualitative and quantitative components of this objective. For the qualitative component, pre-intervention in-depth qualitative data will be collected from three groups of participants (healthcare workers, N=~9; community members, N=~9; patients, N=~12; total N=~30) in each of the regions. A semistructured discussion guide will include discussion questions and participatory activities to elicit knowledge, beliefs and attitudes regarding MDR-TB, and acceptability of the proposed study methodology. This information will be used to inform the design and implementation of the quantitative component of the feasibility study. For the quantitative component, a scoping review on MDR-TB case finding interventions will be conducted to document case finding intervention strategies for MDR-TB in LMICs. The first intervention strategy of the feasibility study, focusing on transmission hotspots, will be tailored to the local context.^12^ For the second intervention strategy of the feasibility study, focusing on household and other contacts, MDR-TB household contact studies from Peru will be used as examples.^13 14^ Peru has a similar TB burden to Namibia, is also an LMIC and has conducted similar case finding studies which serve as good examples for this type of work; we therefore refer to the Peruvian studies as comparison. For the third intervention strategy of the feasibility study, focusing on the TB wards, all consecutive visitors of patients with MDR-TB will be asked to participate (N=~600). We are unaware of similar work of this nature and therefore cannot add comparative data for this strategy.

#### Laboratory analyses

Sputum samples will be collected from enrolled participants for GeneXpert MTB/RIF analysis. Participants with a positive GeneXpert MTB/RIF rifampicin resistant result will be sent to UNAM biosafety level 3 laboratory for culture and drug sensitivity testing. We will collect blood samples for C reactive protein tests and interferon gamma release assays to evaluate latent TB infection. We will have access to a mobile X-ray facility to take participants’ chest X-rays; we will also include chest ultrasounds. HIV rapid tests will be conducted in the field. Positive results from any test will be formally communicated and followed up with the health service providers in the area ensuring that treatment and/or care is initiated.

#### Data management and analysis

Data from the qualitative and quantitative components of the feasibility study will be stored in separate databases. Qualitative data will be captured and analysed with ATLAS.ti using Attride-Stirling’s framework for thematic analysis to determine acceptability.^15^ Feasibility will be determined by collecting data on recruitment and retention, time required to recruit a participant, number of eligible participants required to recruit required sample size and feasibility of testing procedures and data collection methods.^16^ Yield will be determined by using a number-needed-to-screen approach for each strategy.^17^ Cost will be determined using the incremental cost-effectiveness ratio (ICER) for each case finding intervention strategy.^18^

### Objective 3

#### Epidemiological modelling

Modelling will be carried out, premised on data from objectives 1 and 2. Data from objectives 1 and 2 will be used for the construction of a compartmental transmission model calibrated against Namibian data (Census, Demographic and Health Survey, as well as data describing the TB and HIV prevalence, MDR-TB prevalence, TB/MDR-TB case detection and treatment outcomes) that will not only evaluate the MDR-TB epidemic in Namibia but will also investigate the impact of case finding interventions on MDR-TB incidence. In a base scenario, the status quo will be evaluated, assuming no active case finding. Each of the three interventions will then be evaluated and their impact determined though divergence from the base.

#### Health economic modelling

During the feasibility studies, costing data will be collected from each site in a standardised way. Decision analysis will be carried out using the ICER tool. The primary outcome will be the ICER, comparing the three case finding strategies with the baseline scenario, from the societal perspective over a lifetime analytical horizon. One-way sensitivity analyses will be conducted on all model parameters, multiway sensitivity analyses on parameters most critical to the cost-effectiveness estimate, and a probabilistic uncertainty analysis where all parameters are varied simultaneously over distributions to construct 95% uncertainty ranges and cost-effectiveness acceptability curves.

### Ethics approval and consent to participate

**Approvals:** ethics approval was obtained from the UNAM Human Research Ethics Committee for Health (HREC-H) and the Ministry of Health and Social Sciences (MoHSS).

**Regulation:** the UNAM Grants Management Office in collaboration with Professor Mareli Claassens will ensure compliance with requirements for the protection of human subjects in medical research.

**Informed participant consent** will be obtained from all participants before participation. All children and adolescents will be eligible for inclusion in the study. Individuals aged 18+ years will provide consent for themselves. For individuals aged <18 years, consent from parents or legal guardians will be required. For adolescents aged 10–>18 years, following parent/legal guardian consent, additional assent will be requested, developed with information materials and age-appropriate wording in a language of preference. No assent will result in exclusion from the study.

**Participant privacy and protection** will be always respected. Voluntary participation will be emphasised. Test results will be given to participants and communicated to local healthcare facilities for the initiation of TB and/or HIV treatment. Academic reports will be comprised of de-identified data. Hard copies of participant data will be securely safeguarded. Electronic files will be password protected. Sequencing data will be stored without an identifying patient personal profile. Instead, a unique identifier (bar code), will be used in both clinical and sequencing databases thus ensuring privacy. HREC-H, MoHSS and RCB will have access to the data records (clinical, routine laboratory, sequencing) upon request.

Study staff will have to sign confidentiality agreements and undergo Good Clinical Practice training. This will be documented in a regulatory file and stored securely as electronic and hard copies. In addition, there will be comprehensive training on both assenting minors and determining whether they have sufficient capacity to assent. Experts (Professor Seddon and Dr Hoddinott who work at the Desmond Tutu TB Centre in South Africa) will be consulted for the formulation of assent documents. Staff will be trained to exercise extensive precaution to ensure the privacy of minors during and after HIV testing. With a new diagnosis of HIV, staff will link participants to care with appropriate routine health programmes and will ensure the verification thereof.

## Discussion

Our study will provide an improved understanding of the MDR-TB epidemic in Namibia, including its transmission pathways and outbreak cycles. Moreover, it will provide the identification of transmission hotspots to be used for site selection in a targeted case finding feasibility study. Additionally, a model will be developed to evaluate the impact of targeted case finding intervention strategies on MDR-TB incidence. The new technologies used in our study (WGS) will boost cooperation of stakeholders in TB control and support the development of skills and expertise in the country. Furthermore, the findings from our study could be used to allocate programmatic resources according to need (ie, to areas with transmission hotspots), using high-resolution resistance surveillance and transmission data which will guide the NTLP in the development of TB programme guidelines and policies. In addition, the findings could contribute to the design of a large-scale intervention study to find MDR-TB cases that is not only feasible but cost-effective with significant yields and acceptable to communities at large.

We will use the H3TB data in conjunction with other study data, for instance, a study which looked at the ‘Collision of three pandemics: the effect of tuberculosis and HIV on the epidemiological, clinical, virological, and immunological trajectory of Covid-19 in primary healthcare facility attendees (Core-NB)’ funded by the European and Developing Countries Clinical Trials Partnership and conducted in the Omaheke region of Namibia and in Gabarone, Botswana. This study investigated the yield of screening primary healthcare facility attendees, independent of their symptomatology and reason for attending the facility, for active and latent TB. We have completed enrolment and will finalise the analysis within the next few months. Using H3TB Study data in conjunction with Core-NB Study data will give us additional information on the impact of enhanced case finding, not only among patients with DR-TB but also in patients with drug-sensitive TB in our respective contexts. We have been funded by the German government through its ‘Global Health Protection Program’ to acquire a mobile laboratory, kitted with mobile X-ray facility and a MolBio Truenat MTB-RIF Dx. We will use this facility in the H3TB feasibility study and in ongoing projects in the Omaheke region, both of which data could be used in our H3TB dynamic transmission model.

In summary, the H3TB Project will encourage collaborative efforts between community leaders, non-governmental organisations and other stakeholders (NTLP, NIP) to strengthen TB diagnostics, treatment, surveillance and control on numerous levels. In so doing, this will contribute to the building of local and regional capacity and networks to improve early diagnosis and effective treatment of MDR-TB. Findings will formally be disseminated at international meetings such as the International Union against TB and Lung Disease annual conference and in scientific peer-reviewed journals, preferably open access. Social and regular media will be used in addition to informing the lay public about the outcomes.

## Supplementary Material

Reviewer comments

Author's manuscript

## References

[1] Global tuberculosis report 2021 [Internet];

[2] Ruswa N, Mavhunga F, Roscoe JC, et al. Second nationwide anti-tuberculosis drug resistance survey in Namibia. Int J Tuberc Lung Dis 2019;23:858–64. 10.5588/ijtld.18.0526 31439119 PMC6815747

[3] National guidelines for the management of tuberculosis in Searchworks catalog;

[4] COVID-19 pandemic effects hamper communicable diseases fight in Namibia – official | Namibia economist;

[5] Zelner JL, Murray MB, Becerra MC, et al. Identifying Hotspots of Multidrug-Resistant Tuberculosis Transmission Using Spatial and Molecular Genetic Data. J Infect Dis 2016;213:287–94. 10.1093/infdis/jiv387 26175455 PMC4690150

[6] Gardy JL, Johnston JC, Ho Sui SJ, et al. Whole-genome sequencing and social-network analysis of a tuberculosis outbreak. N Engl J Med 2011;364:730–9. 10.1056/NEJMoa1003176 21345102

[7] Merker M, Blin C, Mona S, et al. Evolutionary history and global spread of the Mycobacterium tuberculosis Beijing lineage. Nat Genet 2015;47:242–9. 10.1038/ng.3195 25599400 PMC11044984

[8] Coll F, McNerney R, Guerra-Assunção JA, et al. A robust SNP barcode for typing Mycobacterium tuberculosis complex strains. Nat Commun 2014;5:4812. 10.1038/ncomms5812 25176035 PMC4166679

[9] Walker TM, Ip CLC, Harrell RH, et al. Whole-genome sequencing to delineate Mycobacterium tuberculosis outbreaks: A retrospective observational study. Lancet Infect Dis 2013;13:137–46. 10.1016/S1473-3099(12)70277-3 23158499 PMC3556524

[10] Arain M, Campbell MJ, Cooper CL, et al. What is A pilot or feasibility study? A review of current practice and editorial policy. BMC Med Res Methodol 2010;10:67. 10.1186/1471-2288-10-67 20637084 PMC2912920

[11] Eldridge SM, Lancaster GA, Campbell MJ, et al. Defining Feasibility and Pilot Studies in Preparation for Randomised Controlled Trials: Development of a Conceptual Framework. PLoS One 2016;11:e0150205. 10.1371/journal.pone.0150205 26978655 PMC4792418

[12] Cudahy PGT, Andrews JR, Bilinski A, et al. Spatially targeted screening to reduce tuberculosis transmission in high-incidence settings. The Lancet Infectious Diseases 2019;19:e89–95. 10.1016/S1473-3099(18)30443-2 30554997 PMC6401264

[13] Becerra MC, Appleton SC, Franke MF, et al. Tuberculosis burden in households of patients with multidrug-resistant and extensively drug-resistant tuberculosis: a retrospective cohort study. Lancet 2011;377:147–52. 10.1016/S0140-6736(10)61972-1 21145581 PMC13508782

[14] Grandjean L, Gilman RH, Martin L, et al. Transmission of Multidrug-Resistant and Drug-Susceptible Tuberculosis within Households: A Prospective Cohort Study. PLoS Med 2015;12:e1001843. 10.1371/journal.pmed.1001843 26103620 PMC4477882

[15] Attride-Stirling J. Thematic networks: an analytic tool for qualitative research. Qualitative Research 2001;1:385–405. 10.1177/146879410100100307

[16] Cassidy S, Okwose N, Scragg J, et al. Assessing the feasibility and acceptability of Changing Health for the management of prediabetes: protocol for a pilot study of a digital behavioural intervention. Pilot Feasibility Stud 2019;5. 10.1186/s40814-019-0519-1 PMC6878649 31788325

[17] Naufal F, Chaisson LH, Robsky KO, et al. Number needed to screen for TB in clinical, structural or occupational risk groups. Int J Tuberc Lung Dis 2022;26:500–8. 10.5588/ijtld.21.0749 35650693 PMC9202999

[18] Yadav RP, Nishikiori N, Satha P, et al. Cost-effectiveness of a tuberculosis active case finding program targeting household and neighborhood contacts in Cambodia. Am J Trop Med Hyg 2014;90:866–72. 10.4269/ajtmh.13-0419 24615134 PMC4015580

