# Supplementary material for: Enhanced active case finding of drug-resistant tuberculosis in Namibia: a protocol for the hotspots, hospitals, and households (H3TB) study
Source: BMJ Open. 2024 Feb 10;14(2):e082665. doi: 10.1136/bmjopen-2023-082665 (PMC10862302; doi:10.1136/bmjopen-2023-082665)
Supplement: Author's manuscript [file bmjopen-2023-082665.draft_revisions.pdf]

BMJ Open is committed to open peer review. As part of this commitment we make the peer review history of every article we publish publicly available.

When an article is published we post the peer reviewers' comments and the authors' responses online. We also post the versions of the paper that were used during peer review. These are the versions that the peer review comments apply to.

The versions of the paper that follow are the versions that were submitted during the peer review process. They are not the versions of record or the final published versions. They should not be cited or distributed as the published version of this manuscript.

BMJ Open is an open access journal and the full, final, typeset and author-corrected version of record of the manuscript is available on our site with no access controls, subscription charges or pay-per-view fees (http://bmjopen.bmj.com).

If you have any questions on BMJ Open's open peer review process please email info.bmjopen@bmj.com

BMJ Open

Enhanced active case finding of drug-resistant tuberculosis in Namibia: a protocol for the hotspots, hospitals, and households (H3TB) study

Journal:	*BMJ Open*
Manuscript ID	bmjopen-2023-082665
Article Type:	Protocol
Date Submitted by the Author:	30-Nov-2023
Complete List of Authors:	Shavuka, Olga; University of Namibia, Department of Human Biological and Translational Medical Sciences<br>Iipumbu, Etuhole; University of Namibia, Department of Human Biological and Translational Medical Sciences<br>Boois, Lorraine; University of Namibia, Department of Human Biological and Translational Medical Sciences<br>Haimbala, Victor; University of Namibia, Department of Human Biological and Translational Medical Sciences<br>Günther, Gunar; University of Namibia, Department of Human Biological and Translational Medical Sciences; University of Bern<br>Hoddinott, Graeme; Stellenbosch University Faculty of Medicine and Health Sciences, Desmond Tutu TB Centre<br>Lin, Hsien-Ho; National Taiwan University,<br>Nepolo, Emmanuel; University of Namibia; Inselspital Bern Universitätsklinik für Radio-Onkologie<br>Niemann, Stefan; University of Namibia, Department of Human Biological and Translational Medical Sciences; Forschungszentrum Borstel<br>Ruswa, Nunurai; National Tuberculosis and Leprosy Programme (NTLP)<br>Seddon, James; Imperial College London; Stellenbosch University, Department of Pediatrics and Child Health<br>Claassens, M; Stellenbosch University Faculty of Medicine and Health Sciences, Paediatrics and child health; University of Namibia, Department of Human Biological and Translational Medical Sciences
Keywords:	Tuberculosis < INFECTIOUS DISEASES, Epidemiology < INFECTIOUS DISEASES, Public health < INFECTIOUS DISEASES, Diagnostic microbiology < INFECTIOUS DISEASES

SCHOLARONE™
Manuscripts

Title: Enhanced active case finding of drug-resistant tuberculosis in Namibia: a protocol for the hotspots, hospitals, and households (H3TB) study

Authors: Olga Shavuka[1,#], Etuhole Iipumbu[1,#], Lorraine Boois[1,#], Victor Haimbala[1], Gunar Günther[1,2], Graeme Hoddinott[1,3], Hsien-Ho Lin[4], Emmanuel Nepolo[1], Stefan Niemann[1,5], Nunurai Ruswa[6], James A. Seddon[3,7], Mareli Claassens[1,3,7,&]

# joint first authors

& corresponding author

Affiliations:

1. Department of Human, Biological and Translational Medical Sciences, School of Medicine, Faculty of Health Sciences and Veterinary Medicine, University of Namibia, Windhoek, Namibia
2. Inselspital Bern, University of Bern, Switzerland
3. Desmond Tutu TB Centre, Department of Pediatrics and Child Health, Faculty of Medicine and Health Sciences, Stellenbosch University, Tygerberg, South Africa
4. Institute of Epidemiology and Preventive Medicine, National Taiwan University, Xuzhou Rd, Taipei 100, Taiwan
5. Forschungszentrum Borstel, Parkallee 1, 23845 Borstel, Germany
6. National Tuberculosis and Leprosy Programme (NTLP), Namibia
7. Department of Infectious Disease, Imperial College London, London, United Kingdom

Key Words

Multidrug-Resistant Tuberculosis, Hotspots, Whole Genome Sequencing, Contact Tracing, Community-based Interventions

Word Count

Abstract: 299

Main text: 2807

For peer review only

ABSTRACT

Introduction

Namibia is a high tuberculosis (TB) burden country with an estimated incidence of 460/100,000 (around 12,000 cases) per year. Approximately 4.5% of new cases and 7.9% of previously treated TB cases are multidrug-resistant (MDR) and 47% of MDR-TB patients are HIV co-infected. Published data suggest a clustering of MDR-TB transmission in specific areas. Identifying transmission clusters is key to implementing high yield and cost-effective interventions. This includes knowing the yield of finding TB cases in high transmission zones (e.g., community-hotspots, hospitals, or households) to deliver community-based interventions. We aim to identify such transmission zones for enhanced case finding and evaluate the effectiveness of this approach.

Methods and analysis

H3TB is an observational cross-sectional study evaluating MDR-TB active case finding strategies. Sputum samples from MDR-TB cases in three regions of Namibia will be evaluated by whole genome sequencing (WGS) in addition to routine sputum investigations (Xpert MTB/RIF, culture, and drug susceptibility testing). We will collect information on household contacts, use of community spaces, and geographical map intersections between participants, synthesizing these data to identify transmission hotspots. We will look at the feasibility, acceptability, yield, and cost of case finding strategies in these hotspots, and in households of MDR-TB patients and visitors of hospitalized MDR-TB patients. A compartmental transmission dynamic model will be constructed to evaluate the impact and cost-effectiveness of the strategies if scaled.

Ethics and dissemination

Ethics approval was obtained. Participants will give informed consent. H3TB will capitalize on a partnership with the Ministry of Health and Social Services to follow-up individuals diagnosed with MDR-TB, and integrate WGS data with innovative contact network mapping, to allow enhanced case finding. Study data will contribute towards a systems approach to TB control. Equally important, it will serve as a role model for similar studies in other high incidence settings.

For peer review only

ARTICLE SUMMARY

Strengths and limitations

- State-of-the-art sequencing techniques are implemented and used in a low- and middle-income country (LMIC) setting at the University of Namibia (UNAM).
- Novel DR-TB case finding strategies are piloted and evaluated for feasibility, acceptability, yield, and cost.
- The study is implemented in three Namibian regions which would make the findings generalisable; the transmission model will inform on the impact of scale-up of the case finding strategies in the whole country.
- Existing partnerships between the research group, the National TB, and Leprosy Programme (NTLP), the Research Center Borstel and the Ministry of Health and Social Services could be leveraged to support implementation.
- The study team depends on an existing partnership between UNAM and the NTLP/National Institutes of Pathology (NIP) to obtain sputum culture samples for sequencing; if the routine collection and evaluation of sputum samples do not happen systematically, DR-TB cases might be missed.

INTRODUCTION

Namibia is a classified by the World Health Organization (WHO) as a high-tuberculosis (TB) burden country and, in 2020, the TB incidence was estimated to be 460/100,000, with 12,000 new cases per year (1). The most recent nationwide anti-TB drug resistance survey in Namibia was completed in 2015, reporting a prevalence of multidrug-resistant (MDR)-TB at 4.5% among new cases and 7.9% among previously treated cases. Approximately 46.6% of patients with MDR-TB were coinfected with HIV(2). Based on that prevalence, the number of MDR-TB cases per year is estimated to be around 800. Only 213 cases were detected in 2020.

Namibia faces significant challenges at every step along the TB care cascade, from preventive therapy to diagnostics and patient treatment. Challenges also exist in the identification of prevalent cases of TB infection and disease through passive and active case finding intervention

For peer review only

strategies (3). During the COVID-19 pandemic, Namibia observed an increased number of missed or undiagnosed TB cases with case management interruptions due to the diagnostic supply chain, redirections of resources, and the effects of human movement restrictions, negatively impacting care-seeking (4).

In the 'hotspots, hospitals, and households' (H3TB) study, we aim to identify MDR-TB transmission clusters, through genomic, geospatial, and social data interferences, which could indicate transmission hotspots. We aim to determine the diagnostic yield of targeting these areas for active case finding as well as evaluating the cost effectiveness of such approaches. Our central hypothesis is that geographic heterogeneity in the concentration of genotypes exist within the MDR-TB epidemic in Namibia leading to high transmission areas (hotspots), where MDR-TB incidence could be up to three times that of the baseline population. Further, that if these areas could be identified and targeted through enhanced case finding strategies, these strategies might be more effective than screening and treatment programmes which do not adapt to spatial and biological variation (5). In addition to targeting transmission hotspots, our focus will be on MDR-TB screening of household contacts of MDR-TB cases and visitors of hospitalized MDR-TB patients.

METHODS AND ANALYSIS

Patient and public involvement statement

We have collaborated directly with the NTLP (Dr Nunurai Ruswa, co-author) on the development of this protocol and with the national reference laboratory, the NIP (Ms Queen Mokomele). Since we are a young research group, we have not yet instituted community advisory boards (CAB) to discuss potential studies with community members. However, in a subsequent study we have funding and the expertise to establish a CAB. UNAM research ethics committees will incorporate community members from 2024 onwards.

Design

This is an observational study including the design and evaluation of case finding intervention strategies to determine feasibility. The study has three objectives (**Table 1**). In **Figure 1**, the study

flow is illustrated. Amongst an ongoing parent surveillance study which includes all MDR-TB cases in Namibia from all regions, H3TB is nested within three regions (Khomas, Otjozondjupa and Ohangwena). These regions are known to have a high TB incidence. In areas in the Otjozondjupa region, the case notification rate (CNR) has been >1,000/100,000 (submitted for publication, Claassens). The ongoing parent surveillance study informs the H3TB research team on potential participants to include in Objective 1. In addition, a scoping review looks at existing MDR-TB case finding strategies in low- and middle-income countries (LMIC) and informs Objective 2. Data from the surveillance study and the first two objectives inform the development of the dynamic transmission model, which is Objective 3.

Table 1. Objectives of H3TB study

Objective	Activity
1	To identify transmission hotspots using routine data from the National TB and Leprosy Programme (NTLP), whole genome sequencing (WGS), geospatial and social data from the research study
2	To conduct a feasibility study of three active case finding interventions in three geographical regions
3	To develop a dynamic transmission model to determine the impact of scaled-up interventions on MDR-TB incidence, and evaluate cost-effectiveness

Figure 1 [to be inserted here]. Flow diagram of H3TB study design

Abbreviations: DST: drug sensitivity testing, MDR-TB: multidrug-resistant tuberculosis, p.a.: per annum; black text indicates methods, orange text indicates outcomes

Setting

The study will be conducted in Namibia in three regions, viz., Khomas, Ohangwena and Otjozondjupa. Recruitment has commenced in August 2022 and will continue until December 2024.

Objective 1

For peer review only

Participant Identification

Individuals with a positive Xpert MTB/RIF result with rifampicin resistance demonstrated, and a positive *Mycobacterium tuberculosis (Mtb)* culture, will be included from the three regions. To identify transmission hotspots, whole genome sequencing results will be combined with clinical, epidemiological, geospatial, and social network data. A traditional sample size calculation is not indicated if most of the MDR-TB cases in a region are included (>80%) (5,6). In this study, we might miss some MDR-TB cases who are not reported or diagnosed. However, a current surveillance study which started in January 2020 and is ongoing could inform on additional participants from these areas. We will look at other strategies, like including participants from other regions or finding additional cases through collaborating with the National TB and Leprosy Programme's active case finding interventions, to ensure robustness of our data.

Data Collection

Primary data will be collected using standardized questionnaires. We will use a novel approach of first engaging the participants in an in-depth qualitative interview with participatory data collection activities to facilitate them explaining their family, household, social, and community circumstances. These in-depth qualitative data will facilitate the data collectors' ability to confirm details, probe further, and mitigate a bias toward the under-reporting of contacts. As part of the questionnaires, we will capture a single Global Positioning Satellite (GPS) coordinate for each participant's home and other places in the community which will be identified as "shared spaces" on electronic devices. We will record the coordinate of a participant's home when the field team is at his/her home or at a community space. A separate enrolment form will be used to capture names and other identifying information. Data from the transmission hotspot identification study will be linked to electronic TB register data through personal identifying information such as name, surname, and date of birth. A social network questionnaire will be used to identify shared socialisation settings, with GPS coordinates of these settings recorded.

Laboratory Analyses

DNA will be isolated from sputum cultures at the National Institutes of Pathology (NIP) biosafety level 3 laboratory and the UNAM laboratory for samples with proven GeneXpert® MTB/RIF

For peer review only

rifampicin resistance and shipped to Research Center Borstel (RCB) for WGS. Isolated genomic DNA of individual strains will be sequenced using Illumina sequencing platforms and Nextera library preparation kits. All isolates will be sequenced with a minimum coverage of 50-fold. Raw read data (fastq files) will be mapped to the M. tuberculosis H37Rv genome (GenBank accession number NC_000962.3) using BWA-MEM, and mappings will be refined with the GATK software package. For variant (InDels and SNPs) detection in mapped reads, custom perl scripts will be employed with thresholds of a minimum coverage of 4 reads in both forward and reverse orientation, 4 reads calling the allele with at least a phred score of 20, and 75% allele frequency. Multiple consecutive SNP calls (in a 12 bp window) that could indicate rare recombination sites or reflect artificial variant calls around InDels will be excluded. For alignment of a concatenated sequence for downstream analyses, variants in drug resistance associated genes, and repetitive regions will be excluded. Phylogenetic lineages (Mtb genotypes and known Beijing subgroups) will be inferred from specific SNPs (7,8). As proxy for TB cases associated to direct transmission events, a maximum pairwise genetic distance between at least two Mtb isolates of 5 SNPs will be used (9).

Data Management and Analysis

Data will be captured into databases developed by a data specialist. WGS data will be analysed using a phylogenetic strain classification and a cluster/similarity analysis. Social network data will be analysed using thematic analysis for the description of typical and atypical social network contacts for people with MDR-TB in Namibia. Similarly, thematic analysis will be used for shared space data to identify the types of places where people with MDR-TB interact with other people to whom TB may be transmitted. Social network analysis, in conjunction with WGS cluster data, will indicate transmission hotspots within the regions. In our context, a hotspot is an area with a high TB incidence and where TB transmission happens, based on microbial genomic methods such as WGS and a collection of epidemiologic approaches (social network and spatial analysis) and geographical map intersections between participants.

Objective 2

Participant Identification

For peer review only

For the feasibility study, participants will be enrolled from three zones, namely a) transmission hotspots, b) household and other close contacts of MDR-TB cases identified by the TB programme, and c) visitors to MDR-TB patients admitted to TB wards in hospitals. A sample size calculation is not indicated since one of the goals of feasibility studies is to inform on sample size calculations for the planning of large-scale appropriately powered intervention studies (7,8). A sample size of 600 participants for each intervention strategy will inform on the feasibility and acceptability of the interventions, the yield, and the cost per additional case.

Data Collection

There will be both qualitative and quantitative components of this objective. For the qualitative component, pre-intervention in-depth qualitative data will be collected from three groups of participants (healthcare workers, N~9; community members, N~9; patients, N~12; total N~30) in each of the regions. A semi-structured discussion guide will include discussion questions and participatory activities to elicit knowledge, beliefs and attitudes regarding MDR-TB, and acceptability of the proposed study methodology. This information will be used to inform the design and implementation of the quantitative component of the feasibility study. For the quantitative component a scoping review on MDR-TB case finding interventions will be conducted to document case finding intervention strategies for MDR-TB in low- and middle-income countries. The first intervention strategy of the feasibility study, focusing on transmission hotspots, will be tailored to the local context (12). For the second intervention strategy of the feasibility study, focusing on household and other contacts, MDR-TB household contact studies from Peru will be used as examples (13,14). Peru has a similar TB burden to Namibia, is also a low-/middle income country and has conducted similar case finding studies which serve as good examples for this type of work; we therefore refer to the Peruvian studies as comparison. For the third intervention strategy of the feasibility study, focusing on the TB wards, all consecutive visitors to MDR-TB patients will be asked to participate (N~600). We are unaware of similar work of this nature and therefore cannot add comparative data for this strategy.

Laboratory Analyses

For peer review only

Sputum samples will be collected from enrolled participants for GeneXpert® MTB/RIF analysis. Participants with a positive GeneXpert® MTB/RIF rifampicin resistant result will be sent to UNAM Biosafety Level 3 Laboratory (BSL3) for culture and DST. We will collect blood samples for C-reactive protein tests and interferon gamma release assays (IGRA) to evaluate latent TB infection. We will have access to a mobile x-ray facility to take participants' chest x-rays; we will also include chest ultrasounds. HIV rapid tests will be conducted in the field. Positive results from any test will be formally communicated and followed-up with the health service providers in the area ensuring that treatment and/or care is initiated.

Data Management and Analysis

Data from the qualitative and quantitative components of the feasibility study will be stored in separate databases. Qualitative data will be captured and analysed with ATLAS.ti using Attride-Stirling's framework for thematic analysis to determine acceptability (15). Feasibility will be determined by collecting data on recruitment and retention, time required to recruit a participant, number of eligible participants required to recruit required sample size and feasibility of testing procedures and data collection methods (16). Yield will be determined by using a number needed to screen (NNS) approach for each strategy (17). Cost will be determined using the incremental cost-effectiveness ratio (ICER) for each case finding intervention strategy (18).

Objective 3

Epidemiological Modelling

Modelling will be carried out, premised on data from objectives 1 and 2. Data from objectives 1 and 2 will be used for the construction of a compartmental transmission model calibrated against Namibian data (Census, Demographic and Health Survey, as well as data describing the TB and HIV prevalence, MDR-TB prevalence, TB/ MDR-TB case detection and treatment outcomes) that will not only evaluate the MDR-TB epidemic in Namibia but will also investigate the impact of case finding interventions on MDR-TB incidence. In a base scenario, the status quo will be evaluated, assuming no active case finding. Each of the three interventions will then be evaluated and their impact determined though divergence from the base.

For peer review only

Health Economic Modelling

During the feasibility studies, costing data will be collected from each site in a standardised way. Decision analysis will be carried out using the ICER tool. The primary outcome will be the ICER, comparing the three case-finding strategies with the baseline scenario, from the societal perspective over a lifetime analytic horizon. One-way sensitivity analyses will be conducted on all model parameters, multi-way sensitivity analyses on parameters most critical to the cost-effectiveness estimate, and a probabilistic uncertainty analysis where all parameters are varied simultaneously over distributions to construct 95% uncertainty ranges and cost-effectiveness acceptability curves.

DISCUSSION

Our study will provide an improved understanding of the MDR-TB epidemic in Namibia, including its transmission pathways and outbreak cycles. Moreover, it will provide the identification of transmission hotspots to be used for site selection in a targeted case finding feasibility study. Additionally, a model will be developed to evaluate the impact of targeted case finding intervention strategies on MDR-TB incidence. The new technologies used in our study (WGS) will boost cooperation of stakeholders in TB control and support the development of skills and expertise in the country. Furthermore, the findings from our study could be used to allocate programmatic resources according to need (i.e., to areas with transmission hotspots), using high resolution resistance surveillance and transmission data which will guide the NTLP in the development of TB programme guidelines and policies. In addition, the findings could contribute to the design of a large-scale intervention study to find MDR-TB cases that is not only feasible but cost-effective with significant yields and acceptable to communities at large.

We will use the H3TB data in conjunction with other study data, for instance a study which looked at the “Collision of three pandemics: the effect of tuberculosis and HIV on the epidemiological, clinical, virological, and immunological trajectory of Covid-19 in primary healthcare facility attendees (Core-NB)” funded by the EDCTP and conducted in the Omaheke region of Namibia and in Gabarone, Botswana. This study investigated the yield of screening primary healthcare facility attendees, independent of their symptomatology and reason for attending the facility, for

For peer review only - http://bmjopen.bmj.com/site/about/guidelines.xhtml

active and latent TB. We have completed enrolment and will finalize the analysis within the next few months. Using H3TB study data in conjunction with Core-NB study data will give us additional information on the impact of enhanced case finding, not only amongst DR-TB patients but also in DS-TB patients in our respective contexts. We have been funded by the German government through its "Global Health Protection Program (GHPP)" to acquire a mobile laboratory, kitted with mobile x-ray facility and a MolBio Truenat® MTB-RIF Dx. We will use this facility in the H3TB feasibility study and in ongoing projects in the Omaheke region, both of which data could be used in our H3TB dynamic transmission model.

In summary, the H3TB project will encourage collaborative efforts between community leaders, non-governmental organisations, and other stakeholders (NTLP, NIP) to strengthen TB diagnostics, treatment, surveillance, and control on numerous levels. In so doing, this will contribute to the building of local and regional capacity and networks to improve early diagnosis and effective treatment of MDR-TB. Findings will formally be disseminated at international meetings such as the International Union against TB and Lung Disease annual conference and in scientific peer-reviewed journals, preferably open access. Social and regular media will be used in addition to inform the lay public about the outcomes.

For peer review only

LIST OF ABBREVIATIONS

DNA – Deoxyribonucleic Acid

DRS - Drug Resistance Survey

EDCTP – European and Developing Countries Clinical Trials Partnership

gDST – Genomic drug susceptibility testing

HIV – Human Immunodeficiency Virus

ICER- Incremental Cost-Effectiveness Ratio

MDR – Multidrug resistant

MIRU-VNTR- Mycobacterial Interspersed Repetitive Unit-Variable Number of Tandem Repeat

MOHSS – Ministry of Health and Social Services

MRC – Medical Research Council

MTB – Mycobacterium tuberculosis

NTLP-National TB and Leprosy Programme

NGS – Next Generation Sequencing

TB - Tuberculosis

UNAM – University of Namibia

HREC – Health Research Ethics Committee

WGS – Whole genomic sequencing

WHO - World Health Organization

XDR – Extensively drug resistant

For peer review only

DECLARATIONS

Ethics Approval and Consent to Participate

Approvals: Ethics approval was obtained from the University of Namibia Human Research Ethics Committee for Health (HREC-H) and the Ministry of Health and Social Sciences (MoHSS).

Regulation: The UNAM Grants Management Office in collaboration with Prof. Mareli Claassens will ensure compliance with requirements for the protection of human subjects in medical research.

Informed participant consent: Will be obtained from all participants before participation. All children and adolescents will be eligible for inclusion in the study. Individuals Age 18+: Will provide consent for themselves. Individuals Age <18: Consent from parents or legal guardians will be required. Adolescents aged 10 to >18: Following parent/legal guardian consent, additional assent will be requested, developed with information materials and age-appropriate wording in a language of preference. No assent will result in exclusion from the study.

Participant privacy and protection: Will be always respected. Voluntary participation will be emphasized. Test results will be given to participants and communicated to local healthcare facilities for the initiation of TB and/or HIV treatment. Academic reports will be comprised of de-identified data. Hardcopies of participant data will be securely safeguarded. Electronic files will be password protected. Sequencing data will be stored without an identifying patient personal profile. Instead, a unique identifier (barcode), will be used in both clinical and sequencing databases thus ensuring privacy. HREC-H, MoHSS and RCB will have access to the data records (clinical, routine laboratory, sequencing) upon request.

Study staff: Will have to sign confidentiality agreements and undergo Good Clinical Practice (GCP) training. This will be documented in a regulatory file and stored securely as electronic and hard copies. In addition, there will be comprehensive training on both assenting minors and determining whether they have sufficient capacity to assent. Experts (Prof. Seddon and Dr. Hoddinott that work at the Desmond Tutu TB Centre in South Africa) will be consulted for the formulation of assent documents. Staff will be trained to exercise extensive precaution to ensure the privacy of minors during and after HIV testing. With a new diagnosis of HIV, staff will link

For peer review only

participants to care with appropriate routine health programmes and will ensure the verification thereof.

Author contributions

MC and JS wrote the grant application. EI, LB and OS drafted the protocol manuscript. JS, GH and JS gave detailed input. All other authors contributed to the final version of the manuscript.

Consent for Publication

Not applicable.

Availability of Data and Materials

The stakeholders will be involved in the preparation of manuscripts for international peer reviewed journals under the leadership of the PIs. Papers will be submitted after the approval of all stakeholders and submission of a final report to MOHSS. The data will also be presented at international conferences based on above conditions. Sequencing data will be shared via the global sequencing database Relational Sequencing TB Data Platform (ReSEQTB, https://platform.reseqtb.org).

Competing Interests

The authors declare that they have no competing interests.

Funding

The study is funded by the European and Developing Countries Clinical Trials Partnership (EDCTP) and the British Medical Research Council (MRC). GH receives financial assistance of the European Union (Grant no. DCI-PANAF/2020/420-028), through the African Research Initiative for Scientific Excellence (ARISE), pilot programme. ARISE is implemented by the African Academy of Sciences with support from the European Commission and the African Union Commission. The contents of this document are the sole responsibility of the author(s) and can under no circumstances be regarded as reflecting the position of the European Union, the African Academy of Sciences, and the African Union Commission.

For peer review only

MC is supported jointly by the UK Medical Research Council (MRC) and the UK Foreign, Commonwealth & Development Office (FCDO) under the MRC/FCDO Concordat agreement. This manuscript was produced by H3TB which is part of the EDCTP2 programme supported by the European Union (grant number TMA2018SF-2462). The views and opinions of authors expressed herein do not necessarily state or reflect those of EDCTP.

Acknowledgements

Not applicable.

Registration

The study is registered on Clinicaltrials.gov with number NCT05022238.

For peer review only

REFERENCES

1. Global tuberculosis report 2021 [Internet].

2. Ruswa N, Mavhunga F, Roscoe JC, Beukes A, Shipiki E, van Gorkom J, Sawadogo S, Agolory S, Menzies H, Tiruneh D, Makumbi B, Bayer B, Zezai A, Campbell P, Alexander H, Kalisvaart N, Forster N. Second nationwide anti-tuberculosis drug resistance survey in Namibia. Int J Tuberc Lung Dis. 2019;23(7):858–64.

3. National guidelines for the management of tuberculosis in SearchWorks catalog [Internet].

4. COVID-19 pandemic effects hamper communicable diseases fight in Namibia – Official | Namibia Economist [Internet].

5. Zelner JL, Murray MB, Becerra MC, Galea J, Lecca L, Calderon R, Yataco R, Contreras C, Zhang Z, Manjourides J, Grenfell BT, Cohen T. Identifying Hotspots of Multidrug-Resistant Tuberculosis Transmission Using Spatial and Molecular Genetic Data. J Infect Dis. 2016;213:287–94.

6. Gardy JL, Johnston JC, Sui SJH, Cook VJ, Shah L, Brodkin E, Rempel S, Moore R, Zhao Y, Holt R, Varhol R, Birol I, Lem M, Sharma MK, Elwood K, Jones SJM, Brinkman FSL, Brunham RC, Tang P. Whole-Genome Sequencing and Social-Network Analysis of a Tuberculosis Outbreak. N Engl J Med. 2011;364:730–9.

7. Merker M, Blin C, Wirth T, et al. Evolutionary history and global spread of the Mycobacterium tuberculosis Beijing lineage. Nat Genet. 2015;47(3):242–9.

8. Coll F, McNerney R, Guerra-Assunção JA, Glynn JR, Perdigão J, Viveiros M, Portugal I, Pain A, Martin N, Clark TG. A robust SNP barcode for typing Mycobacterium tuberculosis complex strains. Nat Commun. 2014;5:4812.

9. Walker TM, Ip CLC, Harrell RH, Evans JT, Kapatai G, Dedicoat MJ, Eyre DW, Wilson DJ, Hawkey PM, Crook DW, Parkhill J, Harris D, Walker AS, Bowden R, Monk P, Smith EG, Peto TEA. Whole-genome sequencing to delineate Mycobacterium tuberculosis outbreaks: A retrospective observational study. Lancet Infect Dis. 2013;13:137–46.

10. Arain M, Campbell MJ, Cooper CL, Lancaster GA. What is a pilot or feasibility study? A review of current practice and editorial policy. BMC Medical Research Methodology. 2010;10:67.

11. Eldridge SM, Lancaster GA, Campbell MJ, Thabane L, Hopewell S, Coleman CL, Bond CM. Defining Feasibility and Pilot Studies in Preparation for Randomised Controlled Trials: Development of a Conceptual Framework. PloS ONE. 2016;11(3):e0150205.

For peer review only

12. Cudahy PGT, Andrews JR, Bilinski A, Dowdy DW, Mathema B, Menzies NA, Salomon JA, Shrestha S, Cohen T. Spatially targeted screening to reduce tuberculosis transmission in high-incidence settings. Lancet Infect Dis. 2019;19:e89–95.

13. Becerra MC, Appleton SC, Franke MF, Chalco K, Arteaga F, Bayona J, Murray M, Atwood SS, Mitnick CD. Tuberculosis burden in households of patients with multidrug-resistant and extensively drug-resistant tuberculosis: a retrospective cohort study. Lancet. 2011;377(9760):147–52.

14. Grandjean L, Gilman RH, Martin L, Soto E, Castro B, Lopez S, Coronel J, Castillo E, Alarcon V, Lopez V, Miguel AS, Quispe N, Asencios L, Dye C, Moore DA. Transmission of Multidrug-Resistant and Drug-Susceptible Tuberculosis within Households: A Prospective Cohort Study. PLoS Med. 2015;12(6):e1001843.

15. Attride-Sterling J. Thematic networks: an analytic tool for qualitative research. Qualitative Research. 2001;1(3):385–405.

16. Cassidy S, Okwose N, Scragg J, Houghton D, Ashley K, Trenell MI, Jakovljevic DG, Hallsworth K, Avery L. Assessing the feasibility and acceptability of Changing Health for the management of prediabetes: Protocol for a pilot study of a digital behavioural intervention. Pilot Feasibility Stud. 2019 Nov 26;5(1):1–9.

17. Naufal F, Chaisson LH, Robsky KO, Delgado-Barroso P, Alvarez-Manzo HS, Miller CR, Shapiro AE, Golub JE. Number needed to screen for TB in clinical, structural or occupational risk groups. Int J Tuberc Lung Dis. 2022 Jun 1;26(6):500–8.

18. Yadav RP, Nishikiori N, Satha P, Eang MT, Lubell Y. Cost-Effectiveness of a Tuberculosis Active Case Finding Program Targeting Household and Neighborhood Contacts in Cambodia. Am J Trop Med Hyg. 2014 May 5;90(5):866.

For peer review only

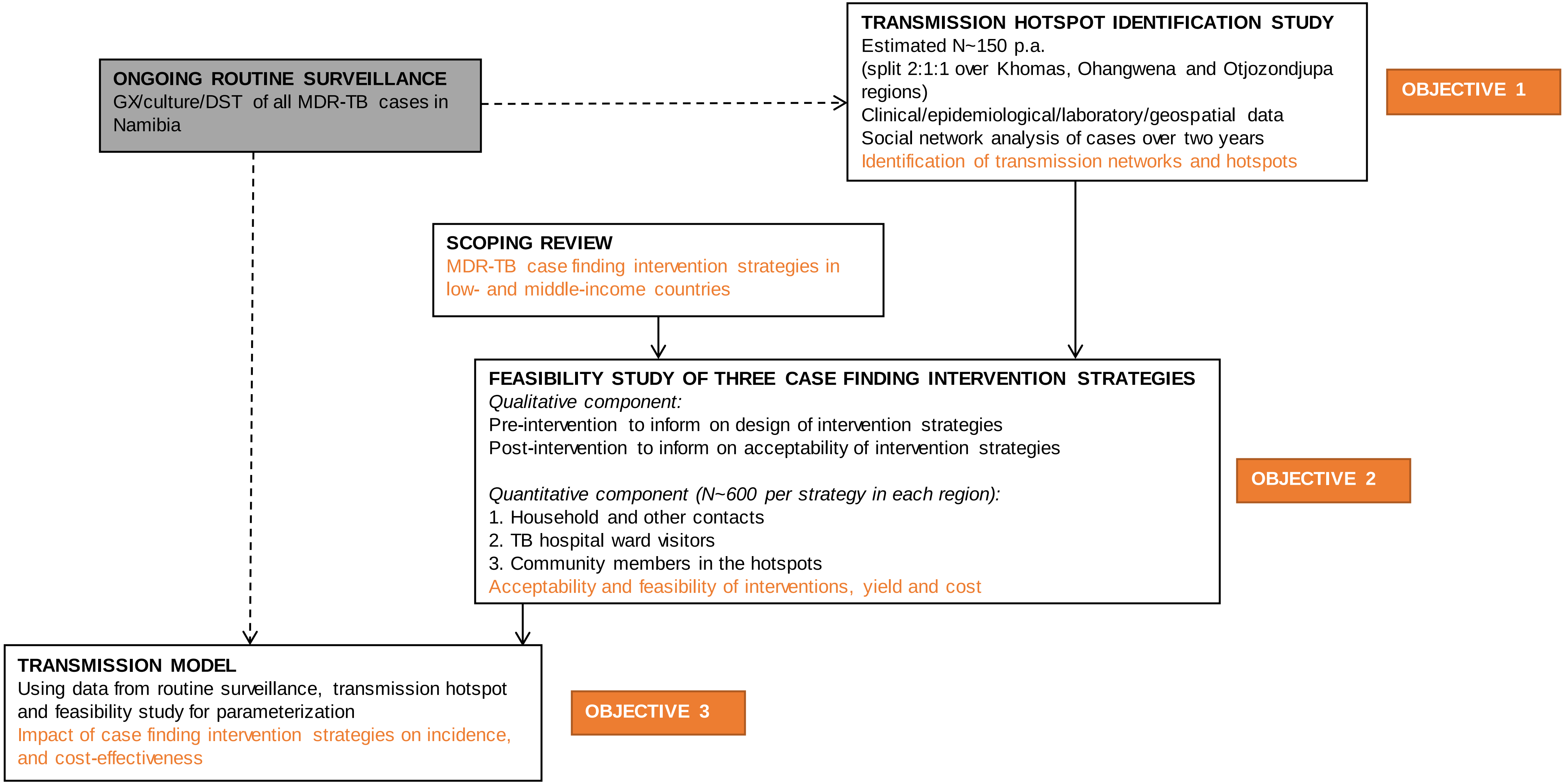
ONGOING ROUTINE SURVEILLANCE
GX/culture/DST of all MDR-TB cases in Namibia
TRANSMISSION HOTSPOT IDENTIFICATION STUDY
Estimated N~150 p.a.
(split 2:1:1 over Khomas, Ohangwena and Otjozondjupa regions)
Clinical/epidemiological/laboratory/geospatial data
Social network analysis of cases over two years
Identification of transmission networks and hotspots
OBJECTIVE 1
SCOPING REVIEW
MDR-TB case finding intervention strategies in low- and middle-income countries
FEASIBILITY STUDY OF THREE CASE FINDING INTERVENTION STRATEGIES
Qualitative component:
Pre-intervention to inform on design of intervention strategies
Post-intervention to inform on acceptability of intervention strategies
Quantitative component (N~600 per strategy in each region):
1. Household and other contacts
2. TB hospital ward visitors
3. Community members in the hotspots
Acceptability and feasibility of interventions, yield and cost
OBJECTIVE 2
TRANSMISSION MODEL
Using data from routine surveillance, transmission hotspot and feasibility study for parameterization
Impact of case finding intervention strategies on incidence, and cost-effectiveness
OBJECTIVE 3

BMJ Open

Enhanced active case finding of drug-resistant tuberculosis in Namibia: a protocol for the hotspots, hospitals, and households (H3TB) study

Journal:	*BMJ Open*
Manuscript ID	bmjopen-2023-082665.R1
Article Type:	Protocol
Date Submitted by the Author:	17-Jan-2024
Complete List of Authors:	Shavuka, Olga; University of Namibia, Department of Human Biological and Translational Medical Sciences<br>Iipumbu, Etuhole; University of Namibia, Department of Human Biological and Translational Medical Sciences<br>Boois, Lorraine; University of Namibia, Department of Human Biological and Translational Medical Sciences<br>Günther, Gunar; University of Namibia, Department of Human Biological and Translational Medical Sciences; University of Bern<br>Hoddinott, Graeme; Stellenbosch University Faculty of Medicine and Health Sciences, Desmond Tutu TB Centre<br>Lin, Hsien-Ho; National Taiwan University,<br>Nepolo, Emmanuel; University of Namibia; Inselspital Bern Universitätsklinik für Radio-Onkologie<br>Niemann, Stefan; University of Namibia, Department of Human Biological and Translational Medical Sciences; Forschungszentrum Borstel<br>Ruswa, Nunurai; National Tuberculosis and Leprosy Programme (NTLP)<br>Seddon, James; Imperial College London; Stellenbosch University, Department of Pediatrics and Child Health<br>Claassens, M; Stellenbosch University Faculty of Medicine and Health Sciences, Paediatrics and child health; University of Namibia, Department of Human Biological and Translational Medical Sciences
**Primary Subject Heading**:	Infectious diseases
Secondary Subject Heading:	Public health, Epidemiology, Respiratory medicine
Keywords:	Tuberculosis < INFECTIOUS DISEASES, Epidemiology < INFECTIOUS DISEASES, Public health < INFECTIOUS DISEASES, Diagnostic microbiology < INFECTIOUS DISEASES

SCHOLARONE™
Manuscripts

Title: Enhanced active case finding of drug-resistant tuberculosis in Namibia: a protocol for the hotspots, hospitals, and households (H3TB) study

Authors: Olga Shavuka[1,#], Etuhole Iipumbu[1,#], Lorraine Boois[1,#], Gunar Günther[1,2], Graeme Hoddinott[1,3], Hsien-Ho Lin[4], Emmanuel Nepolo[1], Stefan Niemann[1,5], Nunurai Ruswa[6], James A. Seddon[3,7], Mareli Claassens[1,3,7,&]

# joint first authors

& corresponding author

Affiliations:

1. Department of Human, Biological and Translational Medical Sciences, School of Medicine, Faculty of Health Sciences and Veterinary Medicine, University of Namibia, Windhoek, Namibia
2. Inselspital Bern, University of Bern, Switzerland
3. Desmond Tutu TB Centre, Department of Pediatrics and Child Health, Faculty of Medicine and Health Sciences, Stellenbosch University, Tygerberg, South Africa
4. Institute of Epidemiology and Preventive Medicine, National Taiwan University, Xuzhou Rd, Taipei 100, Taiwan
5. Forschungszentrum Borstel, Parkallee 1, 23845 Borstel, Germany
6. National Tuberculosis and Leprosy Programme (NTLP), Namibia
7. Department of Infectious Disease, Imperial College London, London, United Kingdom

Key Words

Multidrug-Resistant Tuberculosis, Hotspots, Whole Genome Sequencing, Contact Tracing, Community-based Interventions

Word Count

Abstract: 299

Main text: 2807

Corresponding author: Mareli Claassens, mcla@sun.ac.za

For peer review only

ABSTRACT

Introduction

Namibia is a high tuberculosis (TB) burden country with an estimated incidence of 460/100,000 (around 12,000 cases) per year. Approximately 4.5% of new cases and 7.9% of previously treated TB cases are multidrug-resistant (MDR) and 47% of MDR-TB patients are HIV co-infected. Published data suggest a clustering of MDR-TB transmission in specific areas. Identifying transmission clusters is key to implementing high yield and cost-effective interventions. This includes knowing the yield of finding TB cases in high transmission zones (e.g., community-hotspots, hospitals, or households) to deliver community-based interventions. We aim to identify such transmission zones for enhanced case finding and evaluate the effectiveness of this approach.

Methods and analysis

H3TB is an observational cross-sectional study evaluating MDR-TB active case finding strategies. Sputum samples from MDR-TB cases in three regions of Namibia will be evaluated by whole genome sequencing (WGS) in addition to routine sputum investigations (Xpert MTB/RIF, culture, and drug susceptibility testing). We will collect information on household contacts, use of community spaces, and geographical map intersections between participants, synthesizing these data to identify transmission hotspots. We will look at the feasibility, acceptability, yield, and cost of case finding strategies in these hotspots, and in households of MDR-TB patients and visitors of hospitalized MDR-TB patients. A compartmental transmission dynamic model will be constructed to evaluate the impact and cost-effectiveness of the strategies if scaled.

Ethics and dissemination

Ethics approval was obtained. Participants will give informed consent. H3TB will capitalize on a partnership with the Ministry of Health and Social Services to follow-up individuals diagnosed with MDR-TB, and integrate WGS data with innovative contact network mapping, to allow enhanced case finding. Study data will contribute towards a systems approach to TB control. Equally important, it will serve as a role model for similar studies in other high incidence settings.

For peer review only

ARTICLE SUMMARY

Strengths and limitations

- State-of-the-art sequencing techniques are implemented and used in a low- and middle-income country (LMIC) setting at the University of Namibia (UNAM).
- Novel DR-TB case finding strategies are piloted and evaluated for feasibility, acceptability, yield, and cost.
- The study is implemented in three Namibian regions which would make the findings generalisable; the transmission model will inform on the impact of scale-up of the case finding strategies in the whole country.
- Existing partnerships between the research group, the National TB, and Leprosy Programme (NTLP), the Research Center Borstel and the Ministry of Health and Social Services could be leveraged to support implementation.
- The study team depends on an existing partnership between UNAM and the NTLP/National Institutes of Pathology (NIP) to obtain sputum culture samples for sequencing; if the routine collection and evaluation of sputum samples do not happen systematically, DR-TB cases might be missed.

INTRODUCTION

Namibia is a classified by the World Health Organization (WHO) as a high-tuberculosis (TB) burden country and, in 2020, the TB incidence was estimated to be 460/100,000, with 12,000 new cases per year (1). The most recent nationwide anti-TB drug resistance survey in Namibia was completed in 2015, reporting a prevalence of multidrug-resistant (MDR)-TB at 4.5% among new cases and 7.9% among previously treated cases. Approximately 46.6% of patients with MDR-TB were coinfected with HIV(2). Based on that prevalence, the number of MDR-TB cases per year is estimated to be around 800. Only 213 cases were detected in 2020.

Namibia faces significant challenges at every step along the TB care cascade, from preventive therapy to diagnostics and patient treatment. Challenges also exist in the identification of prevalent cases of TB infection and disease through passive and active case finding intervention

For peer review only

strategies (3). During the COVID-19 pandemic, Namibia observed an increased number of missed or undiagnosed TB cases with case management interruptions due to the diagnostic supply chain, redirections of resources, and the effects of human movement restrictions, negatively impacting care-seeking (4).

In the 'hotspots, hospitals, and households' (H3TB) study, we aim to identify MDR-TB transmission clusters, through genomic, geospatial, and social data interferences, which could indicate transmission hotspots. We aim to determine the diagnostic yield of targeting these areas for active case finding as well as evaluating the cost effectiveness of such approaches. Our central hypothesis is that geographic heterogeneity in the concentration of genotypes exist within the MDR-TB epidemic in Namibia leading to high transmission areas (hotspots), where MDR-TB incidence could be up to three times that of the baseline population. Further, that if these areas could be identified and targeted through enhanced case finding strategies, these strategies might be more effective than screening and treatment programmes which do not adapt to spatial and biological variation (5). In addition to targeting transmission hotspots, our focus will be on MDR-TB screening of household contacts of MDR-TB cases and visitors of hospitalized MDR-TB patients.

METHODS AND ANALYSIS

Patient and public involvement statement

We have collaborated directly with the NTLP (Dr Nunurai Ruswa, co-author) on the development of this protocol and with the national reference laboratory, the NIP (Ms Queen Mokomele). Since we are a young research group, we have not yet instituted community advisory boards (CAB) to discuss potential studies with community members. However, in a subsequent study we have funding and the expertise to establish a CAB. UNAM research ethics committees will incorporate community members from 2024 onwards.

Design

This is an observational study including the design and evaluation of case finding intervention strategies to determine feasibility. The study has three objectives (**Table 1**). In **Figure 1**, the study

For peer review only

flow is illustrated. Amongst an ongoing parent surveillance study which includes all MDR-TB cases in Namibia from all regions, H3TB is nested within three regions (Khomas, Otjozondjupa and Ohangwena). These regions are known to have a high TB incidence. In areas in the Otjozondjupa region, the case notification rate (CNR) has been >1,000/100,000 (submitted for publication, Claassens). The ongoing parent surveillance study informs the H3TB research team on potential participants to include in Objective 1. In addition, a scoping review looks at existing MDR-TB case finding strategies in low- and middle-income countries (LMIC) and informs Objective 2. Data from the surveillance study and the first two objectives inform the development of the dynamic transmission model, which is Objective 3.

Table 1. Objectives of H3TB study

Objective	Activity
1	To identify transmission hotspots using routine data from the National TB and Leprosy Programme (NTLP), whole genome sequencing (WGS), geospatial and social data from the research study
2	To conduct a feasibility study of three active case finding interventions in three geographical regions
3	To develop a dynamic transmission model to determine the impact of scaled-up interventions on MDR-TB incidence, and evaluate cost-effectiveness

Figure 1 [to be inserted here]. Flow diagram of H3TB study design

Abbreviations: DST: drug sensitivity testing, MDR-TB: multidrug-resistant tuberculosis, p.a.: per annum; black text indicates methods, orange text indicates outcomes

Setting

The study will be conducted in Namibia in three regions, viz., Khomas, Ohangwena and Otjozondjupa. Recruitment has commenced in August 2022 and will continue until December 2024.

Objective 1

For peer review only

Participant Identification

Individuals with a positive Xpert MTB/RIF result with rifampicin resistance demonstrated, and a positive *Mycobacterium tuberculosis (Mtb)* culture, will be included from the three regions. To identify transmission hotspots, whole genome sequencing results will be combined with clinical, epidemiological, geospatial, and social network data. A traditional sample size calculation is not indicated if most of the MDR-TB cases in a region are included (>80%) (5,6). In this study, we might miss some MDR-TB cases who are not reported or diagnosed. However, a current surveillance study which started in January 2020 and is ongoing could inform on additional participants from these areas. We will look at other strategies, like including participants from other regions or finding additional cases through collaborating with the National TB and Leprosy Programme's active case finding interventions, to ensure robustness of our data.

Data Collection

Primary data will be collected using standardized questionnaires. We will use a novel approach of first engaging the participants in an in-depth qualitative interview with participatory data collection activities to facilitate them explaining their family, household, social, and community circumstances. These in-depth qualitative data will facilitate the data collectors' ability to confirm details, probe further, and mitigate a bias toward the under-reporting of contacts. As part of the questionnaires, we will capture a single Global Positioning Satellite (GPS) coordinate for each participant's home and other places in the community which will be identified as "shared spaces" on electronic devices. We will record the coordinate of a participant's home when the field team is at his/her home or at a community space. A separate enrolment form will be used to capture names and other identifying information. Data from the transmission hotspot identification study will be linked to electronic TB register data through personal identifying information such as name, surname, and date of birth. A social network questionnaire will be used to identify shared socialisation settings, with GPS coordinates of these settings recorded.

Laboratory Analyses

DNA will be isolated from sputum cultures at the National Institutes of Pathology (NIP) biosafety level 3 laboratory and the UNAM laboratory for samples with proven GeneXpert® MTB/RIF

For peer review only - http://bmjopen.bmj.com/site/about/guidelines.xhtml

rifampicin resistance and shipped to Research Center Borstel (RCB) for WGS. Isolated genomic DNA of individual strains will be sequenced using Illumina sequencing platforms and Nextera library preparation kits. All isolates will be sequenced with a minimum coverage of 50-fold. Raw read data (fastq files) will be mapped to the M. tuberculosis H37Rv genome (GenBank accession number NC_000962.3) using BWA-MEM, and mappings will be refined with the GATK software package. For variant (InDels and SNPs) detection in mapped reads, custom perl scripts will be employed with thresholds of a minimum coverage of 4 reads in both forward and reverse orientation, 4 reads calling the allele with at least a phred score of 20, and 75% allele frequency. Multiple consecutive SNP calls (in a 12 bp window) that could indicate rare recombination sites or reflect artificial variant calls around InDels will be excluded. For alignment of a concatenated sequence for downstream analyses, variants in drug resistance associated genes, and repetitive regions will be excluded. Phylogenetic lineages (Mtb genotypes and known Beijing subgroups) will be inferred from specific SNPs (7,8). As proxy for TB cases associated to direct transmission events, a maximum pairwise genetic distance between at least two Mtb isolates of 5 SNPs will be used (9).

Data Management and Analysis

Data will be captured into databases developed by a data specialist. WGS data will be analysed using a phylogenetic strain classification and a cluster/similarity analysis. Social network data will be analysed using thematic analysis for the description of typical and atypical social network contacts for people with MDR-TB in Namibia. Similarly, thematic analysis will be used for shared space data to identify the types of places where people with MDR-TB interact with other people to whom TB may be transmitted. Social network analysis, in conjunction with WGS cluster data, will indicate transmission hotspots within the regions. In our context, a hotspot is an area with a high TB incidence and where TB transmission happens, based on microbial genomic methods such as WGS and a collection of epidemiologic approaches (social network and spatial analysis) and geographical map intersections between participants.

Objective 2

Participant Identification

For peer review only

For the feasibility study, participants will be enrolled from three zones, namely a) transmission hotspots, b) household and other close contacts of MDR-TB cases identified by the TB programme, and c) visitors to MDR-TB patients admitted to TB wards in hospitals. A sample size calculation is not indicated since one of the goals of feasibility studies is to inform on sample size calculations for the planning of large-scale appropriately powered intervention studies (10,11). A sample size of 600 participants for each intervention strategy will inform on the feasibility and acceptability of the interventions, the yield, and the cost per additional case.

Data Collection

There will be both qualitative and quantitative components of this objective. For the qualitative component, pre-intervention in-depth qualitative data will be collected from three groups of participants (healthcare workers, N~9; community members, N~9; patients, N~12; total N~30) in each of the regions. A semi-structured discussion guide will include discussion questions and participatory activities to elicit knowledge, beliefs and attitudes regarding MDR-TB, and acceptability of the proposed study methodology. This information will be used to inform the design and implementation of the quantitative component of the feasibility study. For the quantitative component a scoping review on MDR-TB case finding interventions will be conducted to document case finding intervention strategies for MDR-TB in low- and middle-income countries. The first intervention strategy of the feasibility study, focusing on transmission hotspots, will be tailored to the local context (12). For the second intervention strategy of the feasibility study, focusing on household and other contacts, MDR-TB household contact studies from Peru will be used as examples (13,14). Peru has a similar TB burden to Namibia, is also a low-/middle income country and has conducted similar case finding studies which serve as good examples for this type of work; we therefore refer to the Peruvian studies as comparison. For the third intervention strategy of the feasibility study, focusing on the TB wards, all consecutive visitors to MDR-TB patients will be asked to participate (N~600). We are unaware of similar work of this nature and therefore cannot add comparative data for this strategy.

Laboratory Analyses

For peer review only

Sputum samples will be collected from enrolled participants for GeneXpert® MTB/RIF analysis. Participants with a positive GeneXpert® MTB/RIF rifampicin resistant result will be sent to UNAM Biosafety Level 3 Laboratory (BSL3) for culture and DST. We will collect blood samples for C-reactive protein tests and interferon gamma release assays (IGRA) to evaluate latent TB infection. We will have access to a mobile x-ray facility to take participants' chest x-rays; we will also include chest ultrasounds. HIV rapid tests will be conducted in the field. Positive results from any test will be formally communicated and followed-up with the health service providers in the area ensuring that treatment and/or care is initiated.

Data Management and Analysis

Data from the qualitative and quantitative components of the feasibility study will be stored in separate databases. Qualitative data will be captured and analysed with ATLAS.ti using Attride-Stirling's framework for thematic analysis to determine acceptability (15). Feasibility will be determined by collecting data on recruitment and retention, time required to recruit a participant, number of eligible participants required to recruit required sample size and feasibility of testing procedures and data collection methods (16). Yield will be determined by using a number needed to screen (NNS) approach for each strategy (17). Cost will be determined using the incremental cost-effectiveness ratio (ICER) for each case finding intervention strategy (18).

Objective 3

Epidemiological Modelling

Modelling will be carried out, premised on data from objectives 1 and 2. Data from objectives 1 and 2 will be used for the construction of a compartmental transmission model calibrated against Namibian data (Census, Demographic and Health Survey, as well as data describing the TB and HIV prevalence, MDR-TB prevalence, TB/ MDR-TB case detection and treatment outcomes) that will not only evaluate the MDR-TB epidemic in Namibia but will also investigate the impact of case finding interventions on MDR-TB incidence. In a base scenario, the status quo will be evaluated, assuming no active case finding. Each of the three interventions will then be evaluated and their impact determined though divergence from the base.

For peer review only

Health Economic Modelling

During the feasibility studies, costing data will be collected from each site in a standardised way. Decision analysis will be carried out using the ICER tool. The primary outcome will be the ICER, comparing the three case-finding strategies with the baseline scenario, from the societal perspective over a lifetime analytic horizon. One-way sensitivity analyses will be conducted on all model parameters, multi-way sensitivity analyses on parameters most critical to the cost-effectiveness estimate, and a probabilistic uncertainty analysis where all parameters are varied simultaneously over distributions to construct 95% uncertainty ranges and cost-effectiveness acceptability curves.

Ethics Approval and Consent to Participate

Approvals: Ethics approval was obtained from the University of Namibia Human Research Ethics Committee for Health (HREC-H) and the Ministry of Health and Social Sciences (MoHSS).

Regulation: The UNAM Grants Management Office in collaboration with Prof. Mareli Claassens will ensure compliance with requirements for the protection of human subjects in medical research.

Informed participant consent: Will be obtained from all participants before participation. All children and adolescents will be eligible for inclusion in the study. Individuals Age 18+: Will provide consent for themselves. Individuals Age <18: Consent from parents or legal guardians will be required. Adolescents aged 10 to >18: Following parent/legal guardian consent, additional assent will be requested, developed with information materials and age-appropriate wording in a language of preference. No assent will result in exclusion from the study.

Participant privacy and protection: Will be always respected. Voluntary participation will be emphasized. Test results will be given to participants and communicated to local healthcare facilities for the initiation of TB and/or HIV treatment. Academic reports will be comprised of de-identified data. Hardcopies of participant data will be securely safeguarded. Electronic files will be password protected. Sequencing data will be stored without an identifying patient personal profile. Instead, a unique identifier (barcode), will be used in both clinical and sequencing

For peer review only

databases thus ensuring privacy. HREC-H, MoHSS and RCB will have access to the data records (clinical, routine laboratory, sequencing) upon request.

Study staff: Will have to sign confidentiality agreements and undergo Good Clinical Practice (GCP) training. This will be documented in a regulatory file and stored securely as electronic and hard copies. In addition, there will be comprehensive training on both assenting minors and determining whether they have sufficient capacity to assent. Experts (Prof. Seddon and Dr. Hoddinott that work at the Desmond Tutu TB Centre in South Africa) will be consulted for the formulation of assent documents. Staff will be trained to exercise extensive precaution to ensure the privacy of minors during and after HIV testing. With a new diagnosis of HIV, staff will link participants to care with appropriate routine health programmes and will ensure the verification thereof.

DISCUSSION

Our study will provide an improved understanding of the MDR-TB epidemic in Namibia, including its transmission pathways and outbreak cycles. Moreover, it will provide the identification of transmission hotspots to be used for site selection in a targeted case finding feasibility study. Additionally, a model will be developed to evaluate the impact of targeted case finding intervention strategies on MDR-TB incidence. The new technologies used in our study (WGS) will boost cooperation of stakeholders in TB control and support the development of skills and expertise in the country. Furthermore, the findings from our study could be used to allocate programmatic resources according to need (i.e., to areas with transmission hotspots), using high resolution resistance surveillance and transmission data which will guide the NTLP in the development of TB programme guidelines and policies. In addition, the findings could contribute to the design of a large-scale intervention study to find MDR-TB cases that is not only feasible but cost-effective with significant yields and acceptable to communities at large.

We will use the H3TB data in conjunction with other study data, for instance a study which looked at the "Collision of three pandemics: the effect of tuberculosis and HIV on the epidemiological, clinical, virological, and immunological trajectory of Covid-19 in primary healthcare facility attendees (Core-NB)" funded by the EDCTP and conducted in the Omaheke region of Namibia

For peer review only

and in Gabarone, Botswana. This study investigated the yield of screening primary healthcare facility attendees, independent of their symptomatology and reason for attending the facility, for active and latent TB. We have completed enrolment and will finalize the analysis within the next few months. Using H3TB study data in conjunction with Core-NB study data will give us additional information on the impact of enhanced case finding, not only amongst DR-TB patients but also in DS-TB patients in our respective contexts. We have been funded by the German government through its "Global Health Protection Program (GHPP)" to acquire a mobile laboratory, kitted with mobile x-ray facility and a MolBio Truenat® MTB-RIF Dx. We will use this facility in the H3TB feasibility study and in ongoing projects in the Omaheke region, both of which data could be used in our H3TB dynamic transmission model.

In summary, the H3TB project will encourage collaborative efforts between community leaders, non-governmental organisations, and other stakeholders (NTLP, NIP) to strengthen TB diagnostics, treatment, surveillance, and control on numerous levels. In so doing, this will contribute to the building of local and regional capacity and networks to improve early diagnosis and effective treatment of MDR-TB. Findings will formally be disseminated at international meetings such as the International Union against TB and Lung Disease annual conference and in scientific peer-reviewed journals, preferably open access. Social and regular media will be used in addition to inform the lay public about the outcomes.

For peer review only

LIST OF ABBREVIATIONS

DNA – Deoxyribonucleic Acid

DRS - Drug Resistance Survey

EDCTP – European and Developing Countries Clinical Trials Partnership

gDST – Genomic drug susceptibility testing

HIV – Human Immunodeficiency Virus

ICER- Incremental Cost-Effectiveness Ratio

MDR – Multidrug resistant

MIRU-VNTR- Mycobacterial Interspersed Repetitive Unit-Variable Number of Tandem Repeat

MOHSS – Ministry of Health and Social Services

MRC – Medical Research Council

MTB – Mycobacterium tuberculosis

NTLP-National TB and Leprosy Programme

NGS – Next Generation Sequencing

TB - Tuberculosis

UNAM – University of Namibia

HREC – Health Research Ethics Committee

WGS – Whole genomic sequencing

WHO - World Health Organization

XDR – Extensively drug resistant

For peer review only

DECLARATIONS

Author contributions

MC, JS, GG, GH, HL, EN, SN and NR conceptualized and designed the study. MC and JS wrote the grant applications. EI, LB, and OS drafted the protocol manuscript. JS, GH and MC gave detailed input to the manuscript. All authors contributed to the final version of the manuscript and agreed to the submission.

Consent for Publication

Not applicable.

Availability of Data and Materials

The stakeholders will be involved in the preparation of manuscripts for international peer reviewed journals under the leadership of the PIs. Papers will be submitted after the approval of all stakeholders and submission of a final report to MOHSS. The data will also be presented at international conferences based on above conditions. Sequencing data will be shared via the global sequencing database Relational Sequencing TB Data Platform (ReSEQTB, https://platform.reseqtb.org).

Competing Interests

The authors declare that they have no competing interests.

Funding

The study is funded by the European and Developing Countries Clinical Trials Partnership (EDCTP) and the British Medical Research Council (MRC). GH receives financial assistance of the European Union (Grant no. DCI-PANAF/2020/420-028), through the African Research Initiative for Scientific Excellence (ARISE), pilot programme. ARISE is implemented by the African Academy of Sciences with support from the European Commission and the African Union Commission. The contents of this document are the sole responsibility of the author(s) and can under no circumstances be regarded as reflecting the position of the European Union, the African Academy of Sciences, and the African Union Commission.

For peer review only

MC is supported jointly by the UK Medical Research Council (MRC) and the UK Foreign, Commonwealth & Development Office (FCDO) under the MRC/FCDO Concordat agreement. This manuscript was produced by H3TB which is part of the EDCTP2 programme supported by the European Union (grant number TMA2018SF-2462). The views and opinions of authors expressed herein do not necessarily state or reflect those of EDCTP.

Acknowledgements

Not applicable.

Registration

The study is registered on Clinicaltrials.gov with number NCT05022238.

For peer review only

REFERENCES

1. Global tuberculosis report 2021 [Internet].

2. Ruswa N, Mavhunga F, Roscoe JC, Beukes A, Shipiki E, van Gorkom J, Sawadogo S, Agolory S, Menzies H, Tiruneh D, Makumbi B, Bayer B, Zezai A, Campbell P, Alexander H, Kalisvaart N, Forster N. Second nationwide anti-tuberculosis drug resistance survey in Namibia. Int J Tuberc Lung Dis. 2019;23(7):858–64.

3. National guidelines for the management of tuberculosis in SearchWorks catalog [Internet].

4. COVID-19 pandemic effects hamper communicable diseases fight in Namibia – Official | Namibia Economist [Internet].

5. Zelner JL, Murray MB, Becerra MC, Galea J, Lecca L, Calderon R, Yataco R, Contreras C, Zhang Z, Manjourides J, Grenfell BT, Cohen T. Identifying Hotspots of Multidrug-Resistant Tuberculosis Transmission Using Spatial and Molecular Genetic Data. J Infect Dis. 2016;213:287–94.

6. Gardy JL, Johnston JC, Sui SJH, Cook VJ, Shah L, Brodkin E, Rempel S, Moore R, Zhao Y, Holt R, Varhol R, Birol I, Lem M, Sharma MK, Elwood K, Jones SJM, Brinkman FSL, Brunham RC, Tang P. Whole-Genome Sequencing and Social-Network Analysis of a Tuberculosis Outbreak. N Engl J Med. 2011;364:730–9.

7. Merker M, Blin C, Wirth T, et al. Evolutionary history and global spread of the Mycobacterium tuberculosis Beijing lineage. Nat Genet. 2015;47(3):242–9.

8. Coll F, McNerney R, Guerra-Assunção JA, Glynn JR, Perdigão J, Viveiros M, Portugal I, Pain A, Martin N, Clark TG. A robust SNP barcode for typing Mycobacterium tuberculosis complex strains. Nat Commun. 2014;5:4812.

9. Walker TM, Ip CLC, Harrell RH, Evans JT, Kapatai G, Dedicoat MJ, Eyre DW, Wilson DJ, Hawkey PM, Crook DW, Parkhill J, Harris D, Walker AS, Bowden R, Monk P, Smith EG, Peto TEA. Whole-genome sequencing to delineate Mycobacterium tuberculosis outbreaks: A retrospective observational study. Lancet Infect Dis. 2013;13:137–46.

10. Arain M, Campbell MJ, Cooper CL, Lancaster GA. What is a pilot or feasibility study? A review of current practice and editorial policy. BMC Med Res Methodol. 2010;10:67.

11. Eldridge SM, Lancaster GA, Campbell MJ, Thabane L, Hopewell S, Coleman CL, Bond CM. Defining Feasibility and Pilot Studies in Preparation for Randomised Controlled Trials: Development of a Conceptual Framework. PLoS One. 2016;11(3):e0150205.

12. Cudahy PGT, Andrews JR, Bilinski A, Dowdy DW, Mathema B, Menzies NA, Salomon JA, Shrestha S, Cohen T. Spatially targeted screening to reduce tuberculosis transmission in high-incidence settings. Lancet Infect Dis. 2019;19:e89–95.

13. Becerra MC, Appleton SC, Franke MF, Chalco K, Arteaga F, Bayona J, Murray M, Atwood SS, Mitnick CD. Tuberculosis burden in households of patients with multidrug-resistant and extensively drug-resistant tuberculosis: a retrospective cohort study. Lancet. 2011;377(9760):147–52.

For peer review only

14. Grandjean L, Gilman RH, Martin L, Soto E, Castro B, Lopez S, Coronel J, Castillo E, Alarcon V, Lopez V, Miguel AS, Quispe N, Asencios L, Dye C, Moore DA. Transmission of Multidrug-Resistant and Drug-Susceptible Tuberculosis within Households: A Prospective Cohort Study. PLoS Med. 2015;12(6):e1001843.

15. Attride-Sterling J. Thematic networks: an analytic tool for qualitative research. Qualitative Research. 2001;1(3):385–405.

16. Cassidy S, Okwose N, Scragg J, Houghton D, Ashley K, Trenell MI, Jakovljevic DG, Hallsworth K, Avery L. Assessing the feasibility and acceptability of Changing Health for the management of prediabetes: Protocol for a pilot study of a digital behavioural intervention. Pilot Feasibility Stud. 2019 Nov 26;5(1):1–9.

17. Naufal F, Chaisson LH, Robsky KO, Delgado-Barroso P, Alvarez-Manzo HS, Miller CR, Shapiro AE, Golub JE. Number needed to screen for TB in clinical, structural or occupational risk groups. Int J Tuberc Lung Dis. 2022 Jun 1;26(6):500–8.

18. Yadav RP, Nishikiori N, Satha P, Eang MT, Lubell Y. Cost-Effectiveness of a Tuberculosis Active Case Finding Program Targeting Household and Neighborhood Contacts in Cambodia. Am J Trop Med Hyg. 2014 May 5;90(5):866.

For peer review only

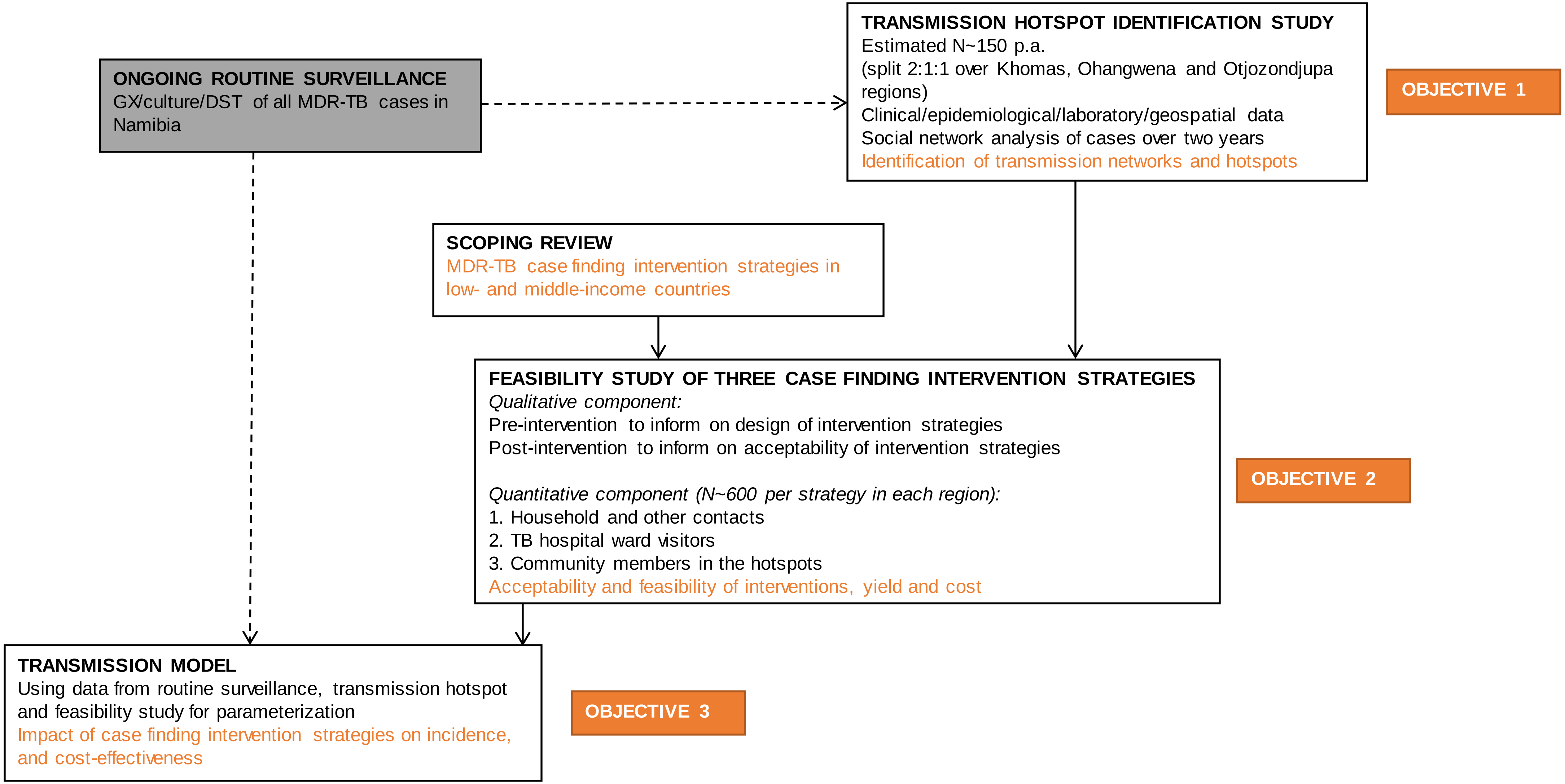
TRANSMISSION HOTSPOT IDENTIFICATION STUDY
Estimated N~150 p.a.
(split 2:1:1 over Khomas, Ohangwena and Otjozondjupa regions)
Clinical/epidemiological/laboratory/geospatial data
Social network analysis of cases over two years
Identification of transmission networks and hotspots
OBJECTIVE 1
ONGOING ROUTINE SURVEILLANCE
GX/culture/DST of all MDR-TB cases in Namibia
SCOPING REVIEW
MDR-TB case finding intervention strategies in low- and middle-income countries
FEASIBILITY STUDY OF THREE CASE FINDING INTERVENTION STRATEGIES
Qualitative component:
Pre-intervention to inform on design of intervention strategies
Post-intervention to inform on acceptability of intervention strategies
Quantitative component (N~600 per strategy in each region):
1. Household and other contacts
2. TB hospital ward visitors
3. Community members in the hotspots
Acceptability and feasibility of interventions, yield and cost
OBJECTIVE 2
TRANSMISSION MODEL
Using data from routine surveillance, transmission hotspot and feasibility study for parameterization
Impact of case finding intervention strategies on incidence, and cost-effectiveness
OBJECTIVE 3
