# Supplementary material for: Enhanced active case finding of drug-resistant tuberculosis in Namibia: a protocol for the hotspots, hospitals, and households (H3TB) study
Source: BMJ Open. 2024 Feb 10;14(2):e082665. doi: 10.1136/bmjopen-2023-082665 (PMC10862302; doi:10.1136/bmjopen-2023-082665)
Supplement: Reviewer comments [file bmjopen-2023-082665.reviewer_comments.pdf]

PEER REVIEW HISTORY

BMJ Open publishes all reviews undertaken for accepted manuscripts. Reviewers are asked to complete a checklist review form (**http://bmjopen.bmj.com/site/about/resources/checklist.pdf**)and are provided with free text boxes to elaborate on their assessment. These free text comments are reproduced below.

ARTICLE DETAILS

**TITLE (PROVISIONAL)**	Enhanced active case finding of drug-resistant tuberculosis in Namibia: a protocol for the hotspots, hospitals, and households (H3TB) study
**AUTHORS**	Shavuka, Olga; Iipumbu, Etuhole; Boois, Lorraine; Günther, Gunar; Hoddinott, Graeme; Lin, Hsien-Ho; Nepolo, Emmanuel; Niemann, Stefan; Ruswa, Nunurai; Seddon, James; Claassens, M

This article was not externally reviewed at BMJ Open. Protocols that have been independently assessed prior to submission to BMJ Open are usually fast-tracked to publication on the grounds that further substantial changes will not be possible. This independent assessment will usually be external, independent review for both a substantial grant award from a non-commercial or government funder and ethics approval.
